# Delayed intra-abdominal bleeding after endoscopic resection of an inverted appendiceal lesion

**DOI:** 10.1055/a-2616-8202

**Published:** 2025-07-02

**Authors:** Takayuki Mori, Yohei Yabuuchi, Kazuya Hosotani, Soichiro Nagao, Shuko Morita, Satoko Inoue, Tetsuro Inokuma

**Affiliations:** 1 26330 Department of Gastroenterology, Kobe City Medical Center General Hospital, Kobe, Japan

An 88-year-old man was referred to our hospital for the treatment of a 40-mm pedunculated polyp located in the cecum ( Fig. 1 ). The lesion was protruding and attached to the tip of the inverted appendix. Endoscopic resection was considered feasible by strangulating the inverted appendix.

**Fig. 1:**
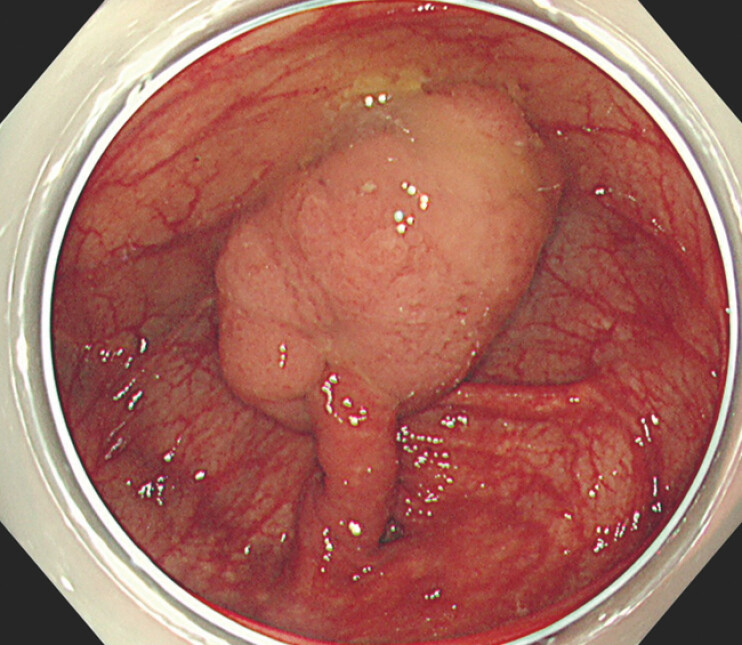
A 40-mm lesion was attached to the tip of the inverted appendix.

The protruding lesion was too large to pass through a detachable snare; therefore, we strangulated the inverted appendix with multiple endoclips and performed endoscopic resection. After complete resection, the defect was closed using additional endoclips. Computed tomography (CT) after the resection confirmed the absence of free air, and the patient was discharged 4 days later. Histological examination revealed that the lesion was completely resected and a tubulovillous adenoma on the inverted appendix was diagnosed ( Fig. 2 ).

**Fig. 2:**
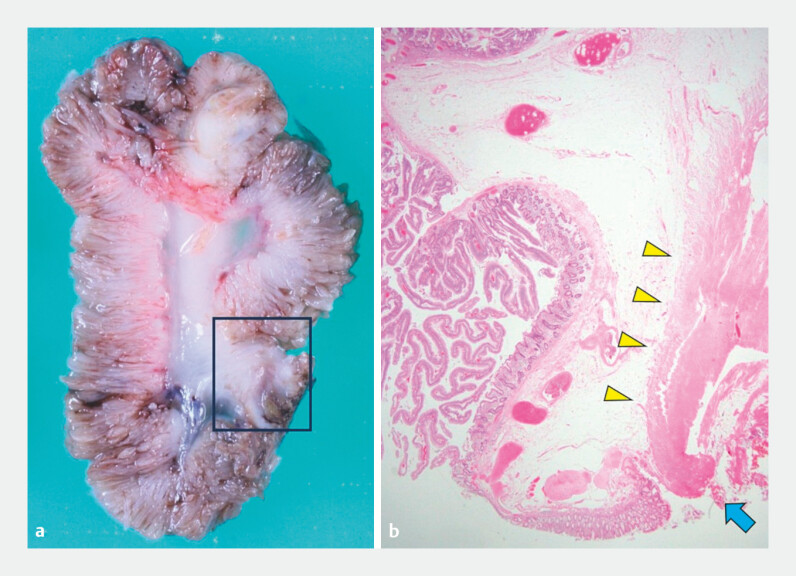
The resected specimen. **a** Cross section of the endoscopically resected specimen. **b** Histological view of the polyp (stained with hematoxylin and eosin), with muscularis propria (yellow arrows), and the cauterized surface at the end of the muscularis propria (blue arrow).

The patient returned to the emergency department of our hospital 13 days after resection, complaining of abdominal pain. CT showed fluid retention around the cecum suspicious for intra-abdominal bleeding, but no free air ( Fig. 3 ). An emergency operation was performed, revealing that the hematoma was due to bleeding from the appendiceal artery ( Fig. 4 ). Cecectomy was performed to achieve hemostasis ( Fig. 5 , Video 1 ).

**Fig. 3:**
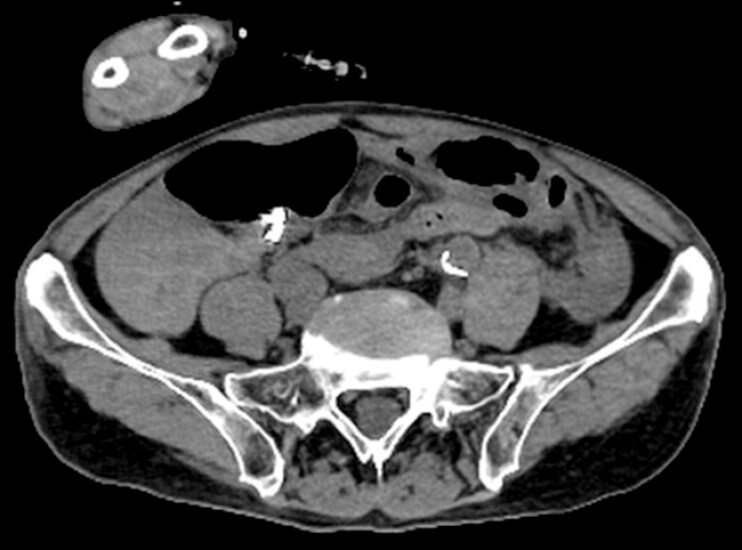
Computed tomography image 13 days after resection.

**Fig. 4:**
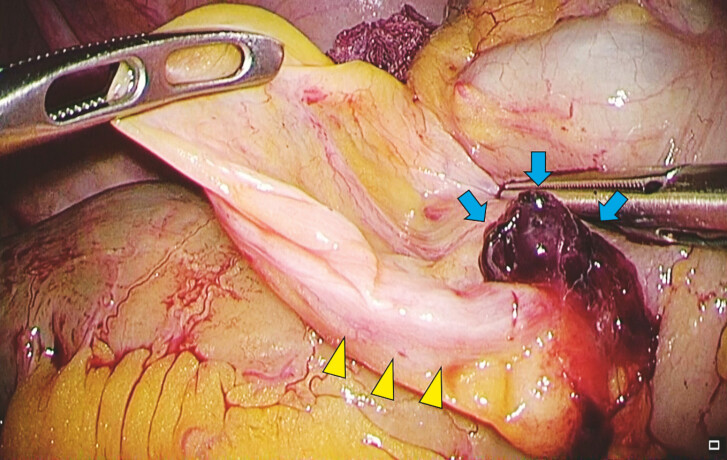
A hematoma (blue arrows) attached to the appendiceal artery (yellow arrows).

**Fig. 5:**
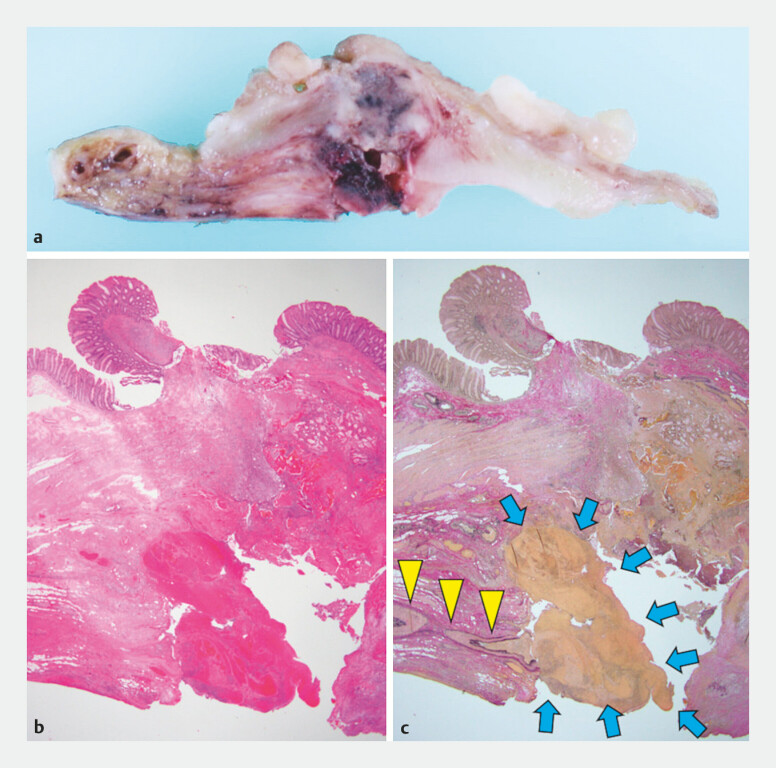
The resected specimen. **a** Cross section of the partially resected cecal specimen. **b** Histological view of the cecum, stained with hematoxylin and eosin. **c** Stained with Elastica van Gieson, a hematoma (blue arrows) attached to the ruptured appendiceal artery (yellow arrows).

Endoscopic resection of a 40-mm pedunculated polyp attached to the tip of the inverted appendix and cecectomy for hemostasis after delayed intra-abdominal bleeding.Video 1

Inverted appendices are extremely rare, with a prevalence of 0.01%; however, they are often associated with lesions at the tip 1 . There have been reports of full-thickness endoscopic resection of inverted appendices with no complications 2 3 . To our knowledge, this is the first report of delayed intra-abdominal bleeding as a complication. In the present case, the inverted appendix was strangulated using endoclips. However, the transected vessel likely leaked into the abdominal cavity leading to delayed intra-abdominal bleeding. When performing resection of an inverted appendix, it is necessary to understand that delayed intra-abdominal bleeding may occur even if strangulation with endoclips is adequately achieved.

Endoscopy_UCTN_Code_CPL_1AJ_2AD_3AD

## Notes

Endoscopy E-Videos https://eref.thieme.de/e-videos
*E-Videos* is an open access online section of the journal *Endoscopy* , reporting on interesting cases and new techniques in gastroenterological endoscopy. All papers include a high-quality video and are published with a Creative Commons CC-BY license. Endoscopy E-Videos qualify for HINARI discounts and waivers and eligibility is automatically checked during the submission process. We grant 100% waivers to articles whose corresponding authors are based in Group A countries and 50% waivers to those who are based in Group B countries as classified by Research4Life (see: https://www.research4life.org/access/eligibility/ ). This section has its own submission website at https://mc.manuscriptcentral.com/e-videos .
